# Anti-hypoxic Effect of Polysaccharide Extract of Brown Seaweed *Sargassum dentifolium* in Tongue Squamous Cell Carcinoma

**DOI:** 10.3389/fnut.2022.854780

**Published:** 2022-03-24

**Authors:** Amira M. Gamal-Eldeen, Bassem M. Raafat, Amani A. Alrehaili, Sherien M. El-Daly, Nahed Hawsawi, Hamsa J. Banjer, Eman M. Raafat, Mazen M. Almehmadi

**Affiliations:** ^1^Clinical Laboratory Sciences Department, College of Applied Medical Sciences, Taif University, Taif, Saudi Arabia; ^2^High Altitude Research Center, Prince Sultan Medical Complex, Al-Hawiyah, Taif University, Taif, Saudi Arabia; ^3^Radiological Sciences Department, College of Applied Medical Sciences, Taif University, Taif, Saudi Arabia; ^4^Medical Biochemistry Department, National Research Centre, Cairo, Egypt; ^5^Cancer Biology and Genetics Laboratory, Centre of Excellence for Advanced Sciences, National Research Centre, Cairo, Egypt; ^6^Department of Pharmacology and Toxicology, Faculty of Pharmacy, Helwan University, Cairo, Egypt

**Keywords:** *Sargassum dentifolium*, miR-21, miR-210, HIF-1α, HIF-1β, CAL-27 OTSCC cells

## Abstract

*Sargassum dentifolium*, (Turner) C. Agarth, 1820, is an edible brown alga collected from red seashores, Egypt. Oral tongue squamous cell carcinoma (OTSCC) is an aggressive malignancy. Hypoxia leads to chemotherapeutic resistance. This work aimed to explore the anti-hypoxia effect of water-soluble polysaccharide fractions of *S. dentifolium* (SD1–SD3) in CAL-27 OTSCC cells. Cell cytotoxicity assay (MTT); cell death mode (DNA staining); total hypoxia (pimonidazole), HIF-1α (ELISA and immunocytochemistry), HIF-1β (ELISA), and hsa-miRNA-21-5p and hsa-miRNA-210-3p (qRT-PCR) were investigated. SD1 and SD2 showed a cytotoxic effect due to apoptosis. SD2 and SD3 decreased total cell hypoxia, inhibited miR-210 (*p* < 0.001 and *p* < 0.01), miR-21 (*p* < 0.01 and *p* < 0.05), and HIF-1α (*p* < 0.01 and *p* < 0.05), respectively. However, only SD3 suppressed HIF-1β (*p* < 0.05). In conclusion, SD2 showed a potential anti-hypoxia effect through amelioration of HIF-1α regulators, which may help in decreasing hypoxia-induced therapeutic resistance.

## Graphical Abstract

Anti-hypoxic activity of *S. dentifolium* through multiple mediators.

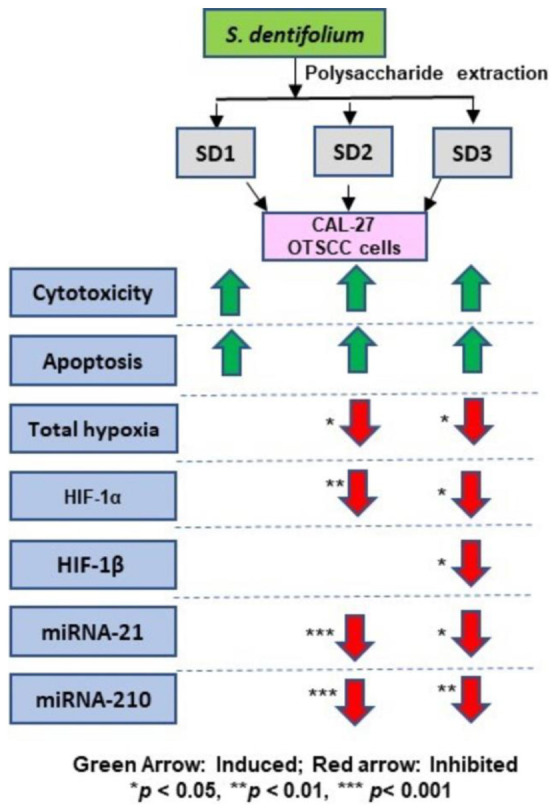

## Introduction

Among brown algae, *Sargassum* is a common tropical/subtropical family that included 150 species (1). A battery of edible *Sargassum* algae that provide a rich source of biologically active polysaccharides, such as *Sargassum stenophyllum*, *Sargassum latifolium*, *Sargassum fulvellum*, and others [reviewed in (2)]. For example, polysaccharide extracts from *Sargassum asperifolium* polysaccharide extracts have been reported to act as tumor anti-initiation activity as well as anti-promotion property *via* their anti-inflammatory activity and a specific anti-progression activity against HepG2 (3). Similarly, the Gamal-Eldeen group (4) reported that *S. latifolium* polysaccharide extracts exhibit promising cancer chemopreventive activity as tumor anti-initiating and anti-promoting agents with specific anti-progression activity against leukemia. Additionally, *Sargassum wightii* polysaccharide extract has been proved to have anti-inflammatory activity (1), antimetastatic activity (5) and a preventive property against oxidative liver injury (6). Moreover, *Sargassum duplicatum* and *S. ilicifolium* in a mixture with other plants have been reported to enhance wound healing in skin (7).

Oral tongue squamous cell carcinoma (OTSCC), as one of the widespread oral cavity malignancies, is a strongly aggressive neoplasm that shows a fast local invasion/spread (8) as a result of a high growth rate and metastasis (9). Nearly 50% of patients have been lately diagnosed in Stages III and IV in the initial diagnostic examination (10). OTSCC has a significant elevated incidence in young to middle-age populations (8), with a poor prognosis, characteristic aggressiveness, and high mortality (19% of patients) (11). OTSCC often leads to functional problems in deglutition, mastication, and speech (12). Although chemotherapy may diminish tumor size and metastasis in OTSCC (12), still, the 5-year survival rate is ∼50% (13).

Hypoxia plays a serious role in the pathophysiology of several human disorders, such as cancer chronic lung disease and ischemic cardiovascular disease (14). In tumor microenvironment (TME), uncontrolled rapid proliferation restricts the oxygen availability (hypoxia) in all solid tumors, where a drop in the normal oxygen (2–9%) down to the hypoxic level <2% occurs (15). The adaption of tumor cells to hypoxia resulted in far aggressive and therapeutically resistant phenotypes. Hypoxia stimulates gene expression alterations followed by consequent proteomic changes that affect cellular and physiological functions (15). Among these changes, cells in TME hypoxic regions divide slowly and escape cytotoxic drugs, which are regularly targeting the rapidly dividing cells (16). In TME, hypoxia produces oxygen gradients that participate in the heterogeneity and plasticity of tumors and evoke aggressive and metastatic phenotypes. During hypoxia, the principal event is the stimulated expression of hypoxia-inducible factor-1α (HIF-1α) that implements a functional role in the cellular consequences prompted in response to hypoxia (17, 18).

Herbal nutraceuticals are functional phytochemicals derived from plants and algae (19). They are nontoxic edible supplements that exhibit broad-spectrum medicinal properties and provide efficient protection against many diseases including cancer (20). Growing studies demonstrate that herbal nutraceuticals could act as safe and effective agents against hypoxic cancer cells *via* successfully attenuating their growth, survival, and progression through the inhibition of HIF-1-signaling pathways (20). Recently, many hypoxia-inhibition strategies have been used as a therapeutic approach to treat cancer through regulating/targeting of HIF-1α to overcome the cell resistance due to hypoxia in solid tumors and through suppressing the hypoxia-stimulated resistance to chemotherapies (21). Among these strategies, using algal and herbal nutraceuticals for targeting downstream HIF-signaling pathways has been done through different approaches, including direct inhibition of HIFs using anticancer agents, blocking the dimerization of HIF-1α and β subunits, and silencing by HIFs-siRNA. Many of herbal and marine agents inhibit the hypoxia-associated resistance to chemotherapies by provoking the activation of an HIF-1α degradation cascade and diminishing the overexpressed HIF-1α in hypoxic tumors [reviewed in (20, 22)]. Extracts of marine algae and sponges from the NCI Open Repository have shown substantial activities as inhibitors for the HIF-1 activation in cell-based assays (23), for example, the red alga *Laurencia intricata* product inhibited HIF-1 activation (24).

In our recent work, we have reported the mechanistic anti-hypoxic role of the *S. latifolium* extract in HCT-116 cells, where the extract has been found to inhibit the hypoxia regulators miRNA-21 and miRNA-210, and consequently suppress HIF-1α and HIF-1β (25). We previously investigated the anti-cancer activity of the polysaccharide extract of the edible alga *S. dentifolium* that indicated its apoptotic activity *via* inducing histone acetylation. Additionally, the extract fractions showed potential anti-genotoxic, anti-mutagenic, and antioxidant activities (26). In the current study, we investigated the influence of *S. dentifolium* polysaccharide extracts on the hypoxia pathway in OTSCC as a continuation of our previous study and due to the lack of studies on *S. dentifolium*, which may help in decreasing hypoxia-induced therapeutic resistance in OTSCC.

## Materials and Methods

### Extraction of *Sargassum dentifolium* Polysaccharides

**FIGURE 1:**
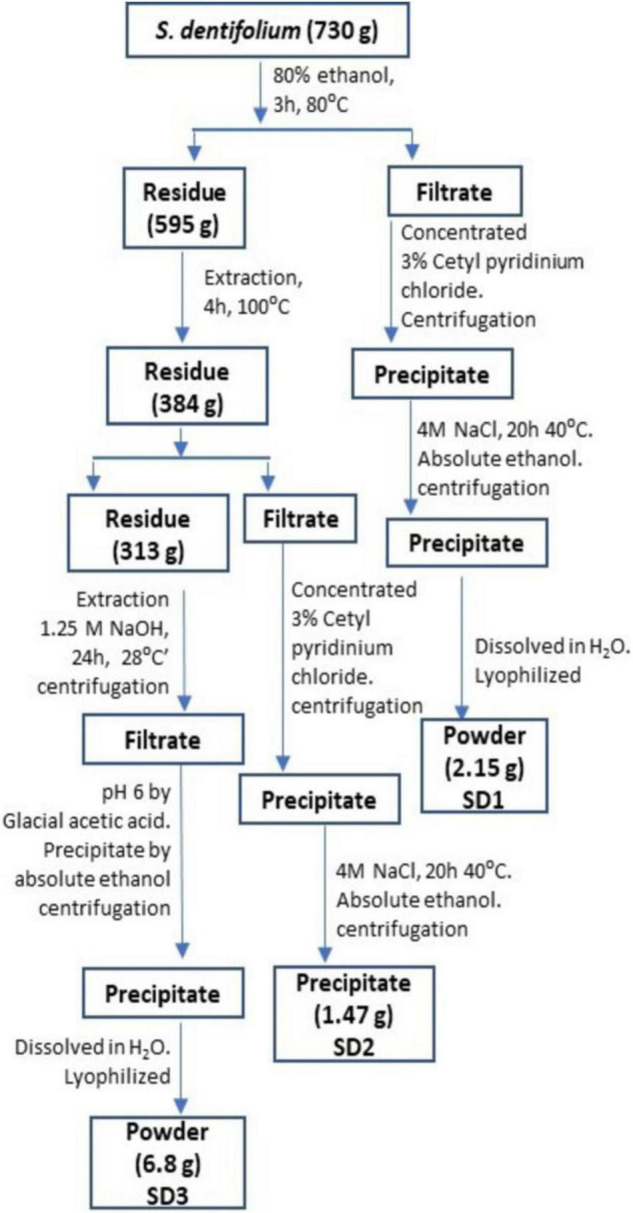
A schematic presentation for extraction protocol and fractionation of *S. dentifolium*.

*Sargassum dentifolium* (Turner) C. Agardh was gathered from Hurghada, Red Sea governorate, Egypt, in December 2018. After several washes and drying, the algal mass was grounded by electric mill and sieved through 2-mm mesh. The extraction of water-soluble polysaccharides has been implemented according to the Zhuang group (27), with some modifications (26), as described in a schematic presentation in Figure 1. The powdered extract fractions (SD1, SD2, and SD3) were investigated for their anti-hypoxic effect.

### Cytotoxicity and Cell Death Mode

Human tongue squamous carcinoma CAL-27 cells (ATCC, United States) were regularly cultured in supplemented DMEM. Chemicals, including celastrol and cell culture materials, were purchased from Sigma–Aldrich (VA, United States), unless mentioned. The cell vitality was tested using a metabolic cytotoxicity MTT assay (28) after 48 h of cell co-culturing with the extract fractions. The data are expressed as % of control cells (mean ± standard deviation). The half-maximal inhibitory concentration (IC_50_) was calculated for the extract fractions. Cells (5 × 10^3^ cells/well) were co-cultured with IC_50_ of fractions, or celastrol, before being stained with ethidium bromide/acridine orange (100 μg/ml; V/V; EB/AO in PBS) (29). The stained cells were analyzed by a fluorescence microscope (*n* = 8; 200×; Axio Imager Z2, Carl Zeiss, Germany).

### Estimation of Total Cellular Hypoxia, HIF-1α, and HIF-1β

Monitoring of the changes in the cellular hypoxia before/after the exposure to extracts was assessed by pimonidazole, a hypoxia-detection reagent, by microplate fluorometer for a qualitative assessment of hypoxia. CAL-27 cells were treated with 30% of IC_50_ of fractions for 6, 12, 24, and 48 h. In another experiment, cells (5 × 10^4^ cells/well) were seeded with 30% of IC_50_ of fractions for 48 h. The cells were lysed by Cell Lysis Solution (#LSKCLS500; Merck, United States) that was supplemented with Protease Inhibitor Cocktail (#P8340; Merck, United States). The cell lysates were then submitted to the analysis with either Human HIF-1α ELISA Fluorescent Kit (#ab229433; Abcam, Germany) or Human ARNT/HIF-1 beta Colorimetric ELISA Kit (#LS-F9594; LifeSpan Biosciences, United States). For the immunocytochemical analysis, CAL-27 cells were cultured onto 8-chamber slides and then treated with 30% of IC_50_ of SD2 for 48 h. The cells were fixed with absolute methanol and then stained using a rabbit monoclonal anti-HIF-1α antibody (Abcam, ab179483), Goat Anti-Rabbit IgG-Phycoerythrin (Abcam, ab72465), and Hoechst 33342 (DNA counterstaining). The cells were analyzed under a fluorescence microscope (Zeiss, Goettingen, Germany), attached to a digital camera (AxioCam MRc3 S/N 4299, Zeiss, Germany) and equipped with an image analyzer (ZEN-11 edition software).

### miR-210 and miR-21 Expression

CAL-27 cells (1 × 10^6^ cells) were treated for 48 h, 30% of IC_50_ of fractions, and then, after, the total RNA was extracted by a miRNeasy RNA extraction kit (#217004, Qiagen, Germany). One microgram of RNA was submitted to reverse transcription by a miScript II RT kit (#218161, Qiagen, Germany). PCR amplification (3-ng cDNA) implemented by a miScript Sybr green PCR kit (#218073, Qiagen, Germany). Primers for U6 (#600750, Agilent technologies), as well as hsa-miR-21 and hsa-miR-210 (#MS00009079 and #MS00003801 Qiagen, Germany), were used. Calculations of the relative miRNA expression were carried out by ΔΔCt protocol (30) after normalization with U6 expression in control cells.

### Statistical Analysis

Experimental analyses were repeated independently (*n* = 6), and the results were presented as mean ± SD. Graphpad Prism software V6 was used. Data were analyzed by Dunnett’s multiple comparisons test after one-way ANOVA. The data were considered significant when *p* < 0.05.

## Results

### Cell Viability and Cell Death Mode

**FIGURE 2:**
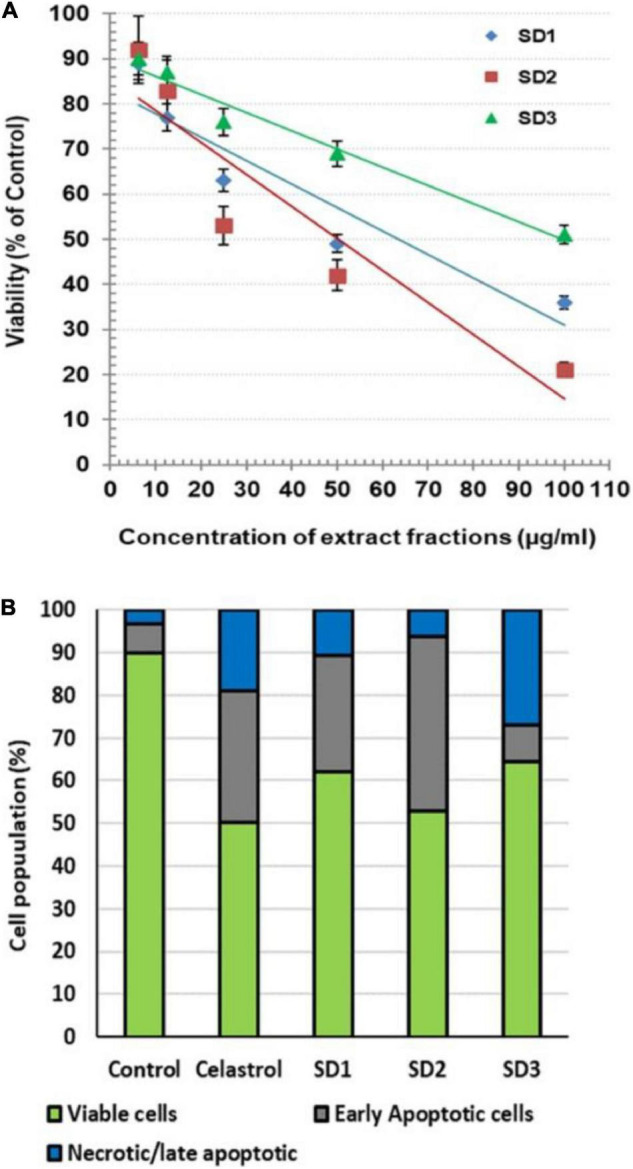
**(A)** Cytotoxic effect of *S dentifolium* extract fractions (SD1, SD2, and SD3) on CAL-27 cells after 48 h of incubation, as assayed by MTT. The viability is expressed as percentage of control (mean ± SD; *n* = 8). **(B)** A segmented histogram indicates the percentage of cell population distribution of vital, early apoptotic, and late apoptotic/necrotic cells after being treated with IC_50_ of celastrol or *S dentifolium* fractions (SD1, SD2, and SD3) for 48 h compared to control.

The possible cytotoxicity effect of the extract fractions, compared with the anticancer drug celastrol, was investigated by MTT after 48 h of co-culturing with CAL-27 cells. The readings showed that the fractions exhibited a variable cytotoxic effect, where they reduced the cell viability with IC_50_ of 63.36, 50.27, and 99.49 μg/ml for SD1, SD2, and SD3, respectively (Figure 2A). Celastrol showed a concentration-dependent cytotoxicity with IC_50_ 8.73 μg/ml. Except in the analysis of a cell death mode, 30% of IC_50_ of each fraction was used in all of the next investigations. The treatment with this IC_50_ percentage leads to a high viability percentage ranged from 84 to 90%, which represents a safe dose to evaluate the protein and genetic changes, as concluded from the concentration/viability equation. AO/EB was used as dual DNA-staining to monitor the cell death mode in CAL-27 cells. After 48 h of incubation with IC_50_ of each agent, the overall analysis of the harvested cells indicated that the treatment with celastrol resulted in a high remarkable increase in the percentages of early apoptotic cells (30.71%; *p* < 0.001) and late apoptotic/necrotic cells (19.12%; *p* < 0.001), compared to their corresponding control, 6.67 and 3.33%, respectively (Figure 2B). In the other hand, the treatment of cells with IC_50_ of fractions led to a noticeable elevation in the early apoptotic population in SD1-treated cells (40.01%; *p* < 0.001) and in SD2-treated cells (27.23%; *p* < 0.001) and a parallel increase in the percentage of late apoptotic/necrotic cells in SD1- and SD2-treated cells (*p* < 0.01 and *p* < 0.05, respectively), as shown in Figure 2B.

### Monitoring of Cellular Hypoxia

**FIGURE 3:**
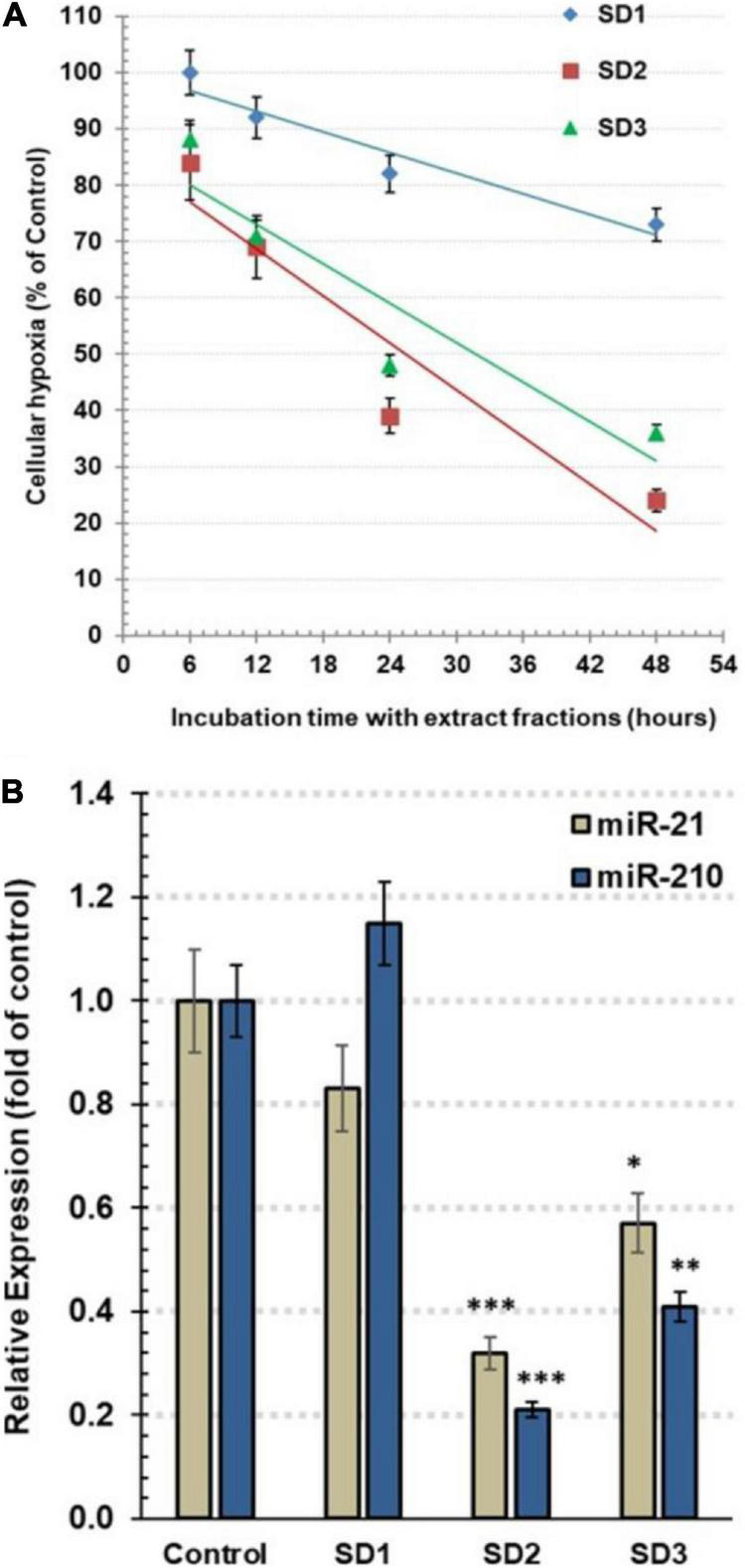
**(A)** Estimation of the total hypoxia degree by pimonidazole in CAL-27 cells. The cells were co-cultured with 30% of IC_50_ of each fraction for 6, 12, 24, and 48 h. The results are expressed as percentage of control (mean ± SD; *n* = 6), where control was 1,374 IFU. **(B)** The relative expression of hsa-miRNA-21-5p and hsa-miRNA-210-3p in CAL-27 cells after being treated with 30% of IC_50_ of fractions for 48 h. Data are expressed as mean ± SD, *n* = 8. *p < 0.05, **p < 0.01, and ***p < 0.001 compared to the corresponding control.

Follow-up of time-dependent alterations in the cellular hypoxia was carried out by pimonidazole, an exogenous nontoxic 2-nitroimidazole. It interacts with the proteins and forms fluorescence adducts, especially with thiol groups, in the hypoxic cells. The fluorescence intensity of these adducts was qualitatively estimated by a microplate fluorescence reader to assess hypoxia occurrence. The cells were co-cultured with 30% of IC_50_ of each fraction for 6, 12, 24, and 48 h. The experiment indicated that SD1 led to a nonsignificant decrease in cell hypoxia (*p* > 0.05), as shown in Figure 3A. In the other hand, SD2 and SD3 resulted in a dramatic time-dependent inhibition in the cell hypoxia, especially after 24 and 48 h (*p* < 0.01; *p* < 0.001, respectively), Figure 3A, where the high maximal inhibitory time for hypoxia (t_50_) for SD2 and SD2 was 25.38 and 31.71 h, respectively.

### miR-210 and miR-21 Expression

To explore the influence of the fractions on the epigenic regulators of cellular hypoxia, the expression of miR-210 and miR-21 was traced by qRT-PCR. As a master hypoxamiR, the oncomiR miR-210 is upregulated, generally, in tumor cells. Perusing of the effect of extract fractions (30% of IC_50_) after 48 h, the results revealed that miR-210 expression was significantly diminished in SD2- and SD3-treated cells, reaching.21 ± 0.03 (*p* < 0.001) and.42 ± 0.06 (*p* < 0.01) fold of the control cells, respectively (Figure 3B). As a potent oncomiR, miR-21 shows a high expression in various tumor cell types. Furthermore, it acts as a pivotal hypoxamiR during a hypoxia cascade. The treatment with 30% of IC_50_ of fractions for 48 h resulted in a dramatic downregulation of miR-21 expression in the cells treated with SD2 and SD3 down to 0.32 ± 0.04 (*p* < 0.01) and 0.57 ± 0.08 (*p* < 0.05) fold of the control cells, respectively (Figure 3B). These findings suggest that SD2 is a potent inhibitor of hypoxia and its regulators.

### Detection of HIF-1α and HIF-1β

**FIGURE 4:**
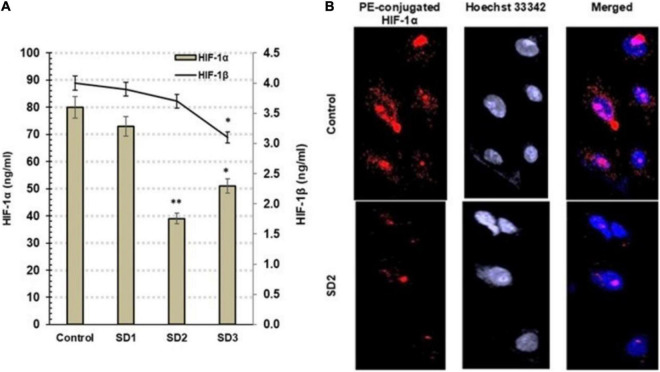
**(A)** Estimation of HIF-1α and HIF-1β by ELISA in CAL-27 cells after being treated with 30% of IC_50_ of each extract fraction for 48 h, compared to control cells, after 48 h. Data are expressed as (mean ± SD in ng/ml, *n* = 6). *p < 0.05, and **p < 0.01 compared with the corresponding control. **(B)** SD2 treatment led to an inhibited HIF-1α; the immunocytochemical staining was implemented by a PE-conjugated HIF-1α antibody (red) and the nuclei counterstaining with DAPI (blue). SD2-treated cells showed a lower HIF-1α concentration than control. The cells were analyzed under fluorescence microscope (magnification ×200).

Hypoxia-inducible factor-1α and HIF-1β are hypoxia indicators that are controlled, generally, by miR-210 and by miR-21 among other miRNAs. Using ELISA, the influence of the fractions on the protein concentrations of HIF-1α and HIF-1β was investigated in CAL-27 cells after 48 h from treatment. Commonly, the elevated HIF-1α is an indication of cellular hypoxia. SD2 was the most potent HIF-1α inhibitor among the tested fractions (*p* < 0.01) after 48 h of treatment (Figure 4A). The immunochemical analysis of the cells confirmed the diminished HIF-1α concentration in SD2-treated cells (Figure 4B), compared to untreated cells.

## Discussion

Herbal nutraceuticals suppress the pathways that induce HIF-1α synthesis and enhance its degradation and reduce its downstream-signaling events (20). Above exerting these anti-cancer activities, herbal nutraceuticals have been reported to (a) reverse hypoxia-stimulated drug resistance, (b) ameliorate the therapeutic index of cancer therapies against hypoxic tumors, and (3) diminish the harmful adverse effects of those therapies. The mechanisms of action of these nutraceuticals and their potent chemo- and radio-sensitization on hypoxic tumor cells are variable and depend on the nutraceuticals type and properties [reviewed in 20, 22]. Therefore, the current study investigated the effect of *S. dentifolium* polysaccharide extracts on the hypoxia pathway in OTSCC, which may provide a new adjacent agent that lowers OTSCC hypoxia-associated resistance to chemotherapies.

Under normoxic conditions, in the ubiquitin-dependent mechanism, O_2_-dependent prolyl hydroxylation targets α subunit of the ubiquitin–proteasome pathway and, hence, degrades HIF-1α (31). Subsequently, HIF-1α remains in a steady state and in a low concentration that prevents the formation of the transcriptional functional complex: HIF-1α/HIF-1β. In the other hand, in severe hypoxic conditions, HIF-1α subunit is mainly the responding part of the HIF-1 complex, and the degradation pathway is inhibited; therefore, HIF-1α concentration is rapidly elevated (31). HIF-1α aggregates in the cytoplasm and then transfers to the nucleus to heterodimerize with the β subunit; binds to hypoxia response elements of target genes, which results in overexpression of hypoxia-regulated genes (14). HIF-1α implicates in the regulation of the cell proliferation, motility, survival, apoptosis, cellular metabolism, and angiogenesis, while the HIF-1β subunit occurs constitutively within cytoplasm. In the current study, exploring the effect of *S. dentifolium* fractions on the cell viability indicated that the fractions exhibited a variable cytotoxic effect on CAL-27 cells in the following order: SD2 > SD1 > SD3. The most potent fraction of the lowest IC_50_ was SD2 due to high percentages of early and late apoptosis.

Hypoxia markers, such as misonidazole, pimonidazole, nitroimidazole, were used to trace cellular the O_2_ profile (32). They have the capability to detect hypoxia due to their production of detectable adducts at low O_2_ tension. For example, pimonidazole is covalently bound to thiol-containing cellular macromolecules in hypoxia cells when the O_2_ tension is below 10-mm Hg, <1.3% (32). In the current study, screening of hypoxia status in CAL-27 cells by pimonidazole after co-culture with *S. dentifolium* fractions for different intervals indicated that SD2 was more potent than SD3 in the time-dependent inhibition of hypoxia, especially after 24 and 48 h. It is known that the regulation of HIF-1α is disturbed as a response to the cellular hypoxia status and/or as a subsequent reflection of genetic alterations that occur in tumor pathophysiology, including tumor invasion, angiogenesis, and cell survival (32). Therefore, exploring the status of HIF-1α protein may be the target mechanism of the extract fraction to inhibit cellular hypoxia. In this study, HIF-1α was found to be basically higher in CAL-27 cells compared to the treated cells. HIF-1α protein was significantly diminished when the cells were treated with SD2 or SD3. The study findings confirmed that SD2 strongly inhibited hypoxia through targeting HIF-1α.

HIF-1β is identical to vertebrate aryl hydrocarbon receptor nuclear translocator (ARNT) (33). It is known that HIF-1β is over-expressed in experimental hypoxic conditions and metastatic tumor cells (34). Contrary to early reports, HIF-1β has a critical function in hypoxic response, where recent reports advocate that HIF-1β concentrations are not consistent during hypoxia. Instead, HIF-1β is fluctuated in response to hypoxic conditions, parallel to HIF-1α, as its heterodimeric partner (34, 35). In the present study, HIF-1β concentration showed a nonsignificant alteration in SD1- and SD2-treated cells, while SD3 treatment led to a noticed inhibition in HIF-1β concentration. Recently, HIF-1β has been reported to be required for full activation of NF-κB in cells in response to canonical and non-canonical stimuli (36), where HIF-1β binds TRAF6 gene and regulates its expression independently from HIF-1α. Moreover, exogenous TRAF6 expression is capable to recover all of the cellular hypoxic phenotypes in absence of HIF-1β. These findings elaborated that HIF-1β is an essential regulator of NF-κB (37). Therefore, the inhibition of HIF-1β by SD3 may provide not only a role in the inhibition of the hypoxia pathway but also a suppression of the NF-κB activation process, a suggestion that needs further investigations.

The majority of miRNAs are alleged to function through RNA-target silencing complexes as a consequence of the impaired base pairing of 30-UTR of certain mRNAs to their target genes; hence, it closes the mRNA translation of these genes or stimulates a direct damaging cleavage (8). A fundamental role is implemented by miRNAs in the upregulation/downregulation of genes in the balance of cell growth/apoptosis (8). Imperfect expression of miRNA often accelerates cancer progression (38). miRNAs are mainly tending to be either oncogenes or tumor suppressors in cancers, including oral cancer (38). OncomiRs, oncogenic miRNAs, are dramatically overexpressed in a way that evoked the neoplastic initiation successively into the tumor progression stage. In the other hand, the tumor suppressive miRNAs play an important role in halting or blocking the tumor development (39). miRNAs expression in OTSCC dominantly controls the activation of gene transcription regulators: protein kinase Ca and HIF-1α that are mainly stimulating the transcription of hypoxamiRs: miR-210 and miR-21 (38). Disturbance of miRNAs in OTSCC resulted in a concomitant induction in cell division, growth rate, anti-apoptosis, metastasis, and drug resistance (38).

miR-210 is an essential responding factor to the hypoxia occurrence in the microenvironment of the endothelial cells, which controls the cells when they are dividing, differentiating, and migrating (40). Previously, fundamental microRNAs functions were recognized in critical cellular pathways in TME. Overexpressed miR-210 was observed to participate in the stabilization of HIF- 1α in TME, in hypoxia (41), and that this overexpression was noticed in head and neck cancerous patients (42). The effect of the extract fractions on the expression of hypoxia-controlling miRNAs (miR-21 and miR-210) was determined. The treatment of OTSCC cells with the fractions SD2 and SD3 dramatically diminished the miR-210 expression. Similarly, the treatment with SD2 and SD3 led to a dramatic down-expression of miR-21 in the cells. miR-21 is overexpressed in most of cancers and possesses several oncogenic functions in carcinomas including OTSCC (43), where miR-21 is correlated with depleted apoptosis (44). Other reports have been reported that using anti-miR-21 to block miR-21 triggers apoptosis and diminishes anchorage-based division of OTSCC cells and likewise suppresses mass xenografts formation of OTSCC in immunocompromised animals (38, 45). A previous report claimed that, in OTSCC, miR-21 decreased the apoptosis through silencing of tropomyosin 1 gene (45). Patients with oral cancer show a successful initial response to chemotherapy; however, a later relapse regularly occurs due to hypoxia-stimulated drug resistance, which limits the chemotherapy effectiveness and its application (46). Therefore, approaches to minimizing the cellular hypoxia through edible complementary agents may facilitate a lower drug resistance and potentiate the efficiency of radio/chemotherapies in oral cancer. Accordingly, the findings of this study deduce *S. dentifolium* extracts as a promising adjacent candidate to chemotherapies for inhibiting hypoxia-induced drug resistance in TME.

## Conclusion

The extract fractions of the edible alga *S. dentifolium* were used in this investigation. Through separate mechanistic amelioration of HIF-1α and HIF-1β and their regulators—miRNA-21 and miRNA-210 - the fractions SD2 and SD3 revealed a possible anti-hypoxia impact in Tongue Squamous Cell Carcinoma (CAL27) cells. The findings imply that *S. dentifolium* could be used as a complementary dietary agent in cancer therapy to reduce hypoxia-related therapeutic resistance in solid tumors.

## Data Availability Statement

The original contributions presented in the study are included in the article/supplementary material, further inquiries can be directed to the corresponding author.

## Author Contributions

AG-E: study concept/design and writing manuscript. BR: statistical analysis. SE-D, NH, AA, HJB, and MA: practical work. ER: alga extraction. All authors contributed to the article and approved the submitted version.

## Conflict of Interest

The authors declare that the research was conducted in the absence of any commercial or financial relationships that could be construed as a potential conflict of interest. The reviewer [AAT] declared a shared affiliation with the authors [SE-D] to the handling editor at the time of review.

## Publisher’s Note

All claims expressed in this article are solely those of the authors and do not necessarily represent those of their affiliated organizations, or those of the publisher, the editors and the reviewers. Any product that may be evaluated in this article, or claim that may be made by its manufacturer, is not guaranteed or endorsed by the publisher.
